# When Sex Doesn't Sell: Using Sexualized Images of Women Reduces Support for Ethical Campaigns

**DOI:** 10.1371/journal.pone.0083311

**Published:** 2013-12-18

**Authors:** Renata Bongiorno, Paul G. Bain, Nick Haslam

**Affiliations:** 1 University of Queensland, Brisbane, Queensland, Australia; 2 University of Melbourne, Melbourne, Victoria, Australia; University of Pennsylvania, United States of America

## Abstract

Images of scantily clad women are used by advertisers to make products more attractive to men. This “sex sells” approach is increasingly employed to promote ethical causes, most prominently by the animal-rights organization PETA. Yet sexualized images can dehumanize women, leaving an unresolved paradox – is it effective to advertise an ethical cause using unethical means? In Study 1, a sample of Australian male undergraduates (N = 82) viewed PETA advertisements containing either sexualized or non-sexualized images of women. Intentions to support the ethical organization were reduced for those exposed to the sexualized advertising, and this was explained by their dehumanization of the sexualized women, and not by increased arousal. Study 2 used a mixed-gender community sample from the United States (N = 280), replicating this finding and extending it by showing that behaviors helpful to the ethical cause diminished after viewing the sexualized advertisements, which was again mediated by the dehumanization of the women depicted. Alternative explanations relating to the reduced credibility of the sexualized women and their objectification were not supported. When promoting ethical causes, organizations may benefit from using advertising strategies that do not dehumanize women.

## Introduction

It is a truism that “sex sells”. Advertising images of scantily clad women aim to arouse men so that their positive reaction becomes associated with a product [1], [2]. Although the use of these images has been criticized as sexist and unethical [2], they are increasingly employed to promote ethical causes. Most prominent is the animal-rights organization PETA (People for the Ethical Treatment of Animals), which regularly uses sexualized images of women in its advertisements. Although sex may sell products, we examined experimentally whether it can sell such ethical causes.

Men support animal-rights less than women [3], [4], so it is understandable for animal-rights campaigners to target men and to use the “sex sells” approach. Sexualized images of women in advertising are widespread [5], [6], [7] and expectations of their positive effects on associated products are typically attributed to heterosexual men's greater arousal and approach tendencies towards sexualized women [8], [9], [10]. Research has shown that the sex sells strategy is generally effective [11], with its use linked to increased purchasing intentions for a wide range of consumer products, including suntan lotion [12], alcohol and jeans [13], and sports shoes [14].

Remarkably, very little research has examined whether the sex sells effect is moderated by perceptions of its relevance or appropriateness for certain products [11], [15], with the apparent view amongst advertising practitioners that it can be used to sell anything [15]. However, when it comes to selling ethical causes – which we define as those causes promoting concern for the welfare of others – the dark side of sexualized advertising may limit its effectiveness.

Research has shown that sexualized women are dehumanized, specifically being seen as more animal-like than non-sexualized women [10]. This subtle form of dehumanization, called infrahumanization [16], involves seeing another as lacking uniquely human characteristics such as rationality, refinement, and culture [17]. Dehumanization can have damaging consequences for its targets [18], [19]. For example, men who dehumanize women by associating them with animals or objects are more likely to sexually harass women and have a higher rape propensity [19].

Sexualized advertising could therefore backfire for ethical causes by eliciting responses that are antithetical to such causes. That is, a cause that seeks to increase moral concern for some living things, such as animals, is inconsistent with and likely to be undermined by sexualized imagery that diminishes moral concern for others (e.g., by dehumanizing women). Several theorists have argued that moral concern for women and animals are closely linked [20], [21], and this link may underpin the inconsistency of promoting the moral value of animals by morally devaluing women.

We hypothesized that sexualized advertisements for an ethical cause would actually *decrease* intentions and behaviors helpful to the cause by encouraging the dehumanization of the women depicted. This mediation hypothesis was supported across two studies, and alternative explanations of increased arousal (Study 1); or the reduced credibility or objectification of the women in the sexualized advertising (Study 2), were not supported.

## Study 1

Our first study provided the initial test of our hypothesis, using a sample of men to test our dehumanization predictions against the typical “sex-sells” effect. We hypothesized that men exposed to sexualized advertisements for an ethical organization would show lower intentions to support it than men exposed to non-sexualized advertisements. We further hypothesized that this effect would be mediated by the dehumanization of the women depicted in the sexualized advertisements. Men's sexual arousal was examined as an alternative mediator.

### Method and Materials

The study was administered online and approved by the Ethical Review Committee of the University of Queensland. As it is impractical to obtain written consent for online surveys, consent was demonstrated at two points: (i) clicking an “I agree” button at the end of the online consent form; and (ii) submission of the completed survey.

Ninety-six self-identified males enrolled as undergraduates in an Australian university (*M*_age_ = 21.33, *SD* = 4.66) were randomly assigned by a computer program to “sexualized” or “non-sexualized” conditions, viewing three advertisements from PETA matched on campaign, depicting women either in lingerie/nude, or fully-clothed (see Appendix S1). Participants rated their degree of arousal for each advertisement (α = .90; “Do you find this advertisement arousing?”; 1 = *Not at all*, 7 = *Very much*) embedded among distractor items (e.g., “Do you like this advertisement?”). Participants then rated the uniquely human (UH) characteristics of the women in the advertisements, using a six-item scale [17] (α = .88; e.g., “civilized”, “refined”; 1 = *Not at all*, 10 = *Very much*), with lower ratings indicating dehumanization. Finally, participants indicated their behavioral intentions to support PETA using a four-item scale (α = .89; e.g., sign a PETA petition, participate in actions organized by PETA; 1 = *Strongly disagree*, 7 = *Strongly agree*; see Appendix S2 for the full list of items)

### Results and Discussion

#### Preliminary analyses

As the advertisements were designed to influence people to behave in ways supporting animal rights, six people who indicated they were already vegetarian or vegan were excluded. A further three people were excluded as a suspicion check revealed they had correctly guessed the experimental aims (e.g., “to assess the impact of the sexualization of a moral issue”). Finally, five participants who were identified in a boxplot as extreme cases in the time taken to complete the short online survey (30–148 minutes) were excluded, as this strongly suggested they did not complete the survey in a single sitting. This resulted in a final sample of 82 (*M*_age_ = 20.70, *SD* = 3.80).

#### Main analyses

**Figure 1:**
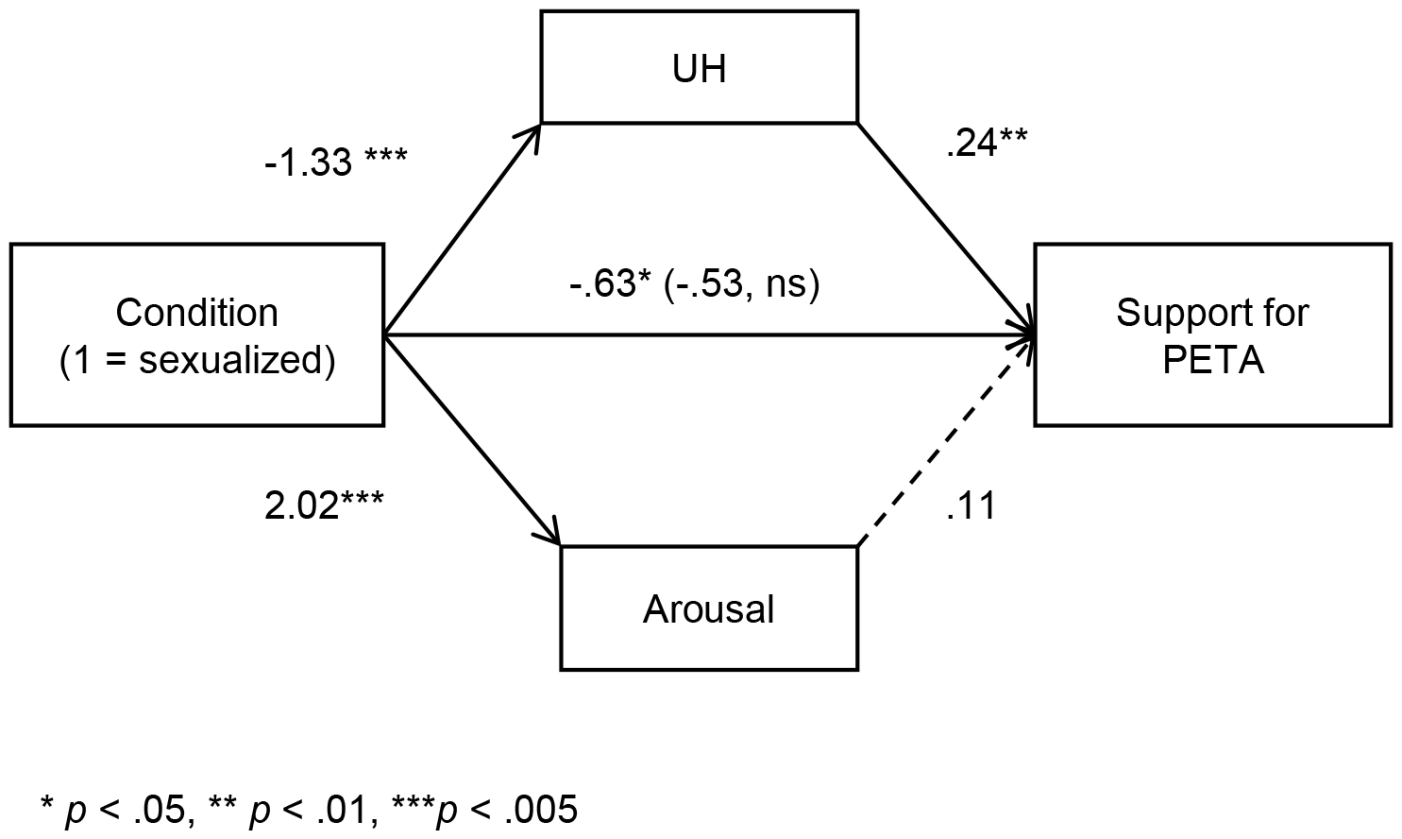
The impact of sexualized advertising on intentions to support PETA (Study 1). Mediation model showing the effect of sexualized images on support for PETA mediated by perceptions of humanness (UH) and arousal. Solid lines represent significant paths, dashed lines represent non-significant paths.

Results confirmed predictions, with support for PETA lower in the sexualized (*M* = 2.65, *SD* = 1.31) than in the non-sexualized (*M* = 3.28, *SD* = 1.39) condition, *t*(80) = 2.11, *p* = .038. Multiple mediation was conducted with UH and arousal scales as parallel mediators, using Hayes' “Process” bootstrap macro for SPSS (5000 resamples)[22]. The mediation model is shown in Figure 1, revealing that while men found the sexualized advertisements both more arousing and more dehumanizing (lower UH), dehumanization was significantly negatively related to support for PETA whereas arousal was not significantly related to support for PETA. Only the mediation effect through dehumanization (lower UH) was significant, indicated by a 95% confidence interval not including zero (UH: [−.69, −.10]; arousal: [−.18, .72]; see Table 1 for means and correlations between items). That is, male participants showed reduced intentions to support PETA after viewing the sexualized advertising, and this relationship was explained by their dehumanization of the women in the advertisements, but not by their arousal (for analyses examining political orientation and age, see Analyses S1).

**Table 1:** Study 1 Means (SDs) for Non-Sexualized and Sexualized Conditions and Correlations for PETA Support, UH, and Arousal.

Study 1	Non-sexualized	Sexualized	1.	2.
1. PETA support	3.28 (1.39)	2.65 (1.31)	–	
2. UH	6.14 (1.60)	4.81 (1.85)	.37**	–
3. Arousal	2.32 (1.41)	4.34 (1.66)	.00	−.11

*p*<.05.

** *p*<.01.

## Study 2

Having ruled out effects for arousal using a sample of male undergraduates, Study 2 involved a larger-scale replication using a mixed-gender community sample from a different country to Study 1, and a larger sample of advertisements. Besides dehumanization, the related but distinct concept of objectification – focusing on a woman's body to the exclusion of her other qualities [23] – was included as another potential mediator. Objectification research has demonstrated that sexualized women receive greater focus on their appearance [24] and are attributed lesser agency [25], [26], competence [27], moral status and mental capacity [28]. Thus, to the extent that sexualized advertisements increase objectification of the women, this could also undermine the ethical message, reducing support. Credibility was also included as a potential mediator, as reduced support might also stem from the sexualized women being seen as less believable message sources than the non-sexualized women. Moreover, the study included an additional behavioral measure of support. Finally, as it might be argued that results showing decreased support for PETA in Study 1 were influenced by participants having already rated targets on the dehumanization-related traits, we reversed the order by obtaining measures of support for PETA before obtaining UH and other ratings.

### Method and Materials

A community sample consisting of 329 people in the United States (159 men, 170 women; self-identified; *M*_age_ = 31.57, *SD* = 10.95) recruited through Mechanical Turk [29]. This study used six PETA advertisements in each condition, again matched on campaign (see Appendix S1). Ethics approval and participant consent was obtained in the same way as in Study 1.

After viewing the advertisements, participants first rated their intentions to support PETA using the scale from Study 1, with the addition of a reverse-worded item (5 items, α = .90). As an additional behavioral measure, participants were then asked to list their ideas for ways to raise awareness and concern for animals, with space for up to four ideas. Following this, participants rated the women in the advertisements on UH using Study 1 items (α = .92), and objectification, using five agency-based items assessing attributions of “mind” [25] (α = .89; e.g., “Compared to the average person, to what extent do you believe the people in these advertisements are capable of: thought, memory?”; 1 = *Much less capable*, 3 = *Equally capable*, 5 = *Much more capable*). Finally, participants rated the credibility of women in the advertisements using six items (α = .94; e.g., “Overall, to what extent do you believe the people in these advertisements are: genuine supporters of animal rights, credible representatives for the animal-rights cause?”; 1 = *Not at all*, 7 = *Very much*; for a complete list of items used, see Appendix S2)

### Results and Discussion

#### Preliminary analyses

Following the same procedures as Study 1, 39 people who indicated they were already vegetarian or vegan were excluded. A further four people were excluded as a debriefing question revealed they had correctly guessed the experimental aims (e.g., “To find out people's feelings on ethical treatment of animals in relation to their feelings on ethical treatment of other people”). Six people identified in a boxplot as extreme cases in the time taken to complete the short online survey (38–111 minutes) were also excluded. This resulted in a final sample of 280 (144 men, 136 women; self-identified; *M*_age_ = 31.65, *SD* = 11.07).

#### Main analyses

**Figure 2:**
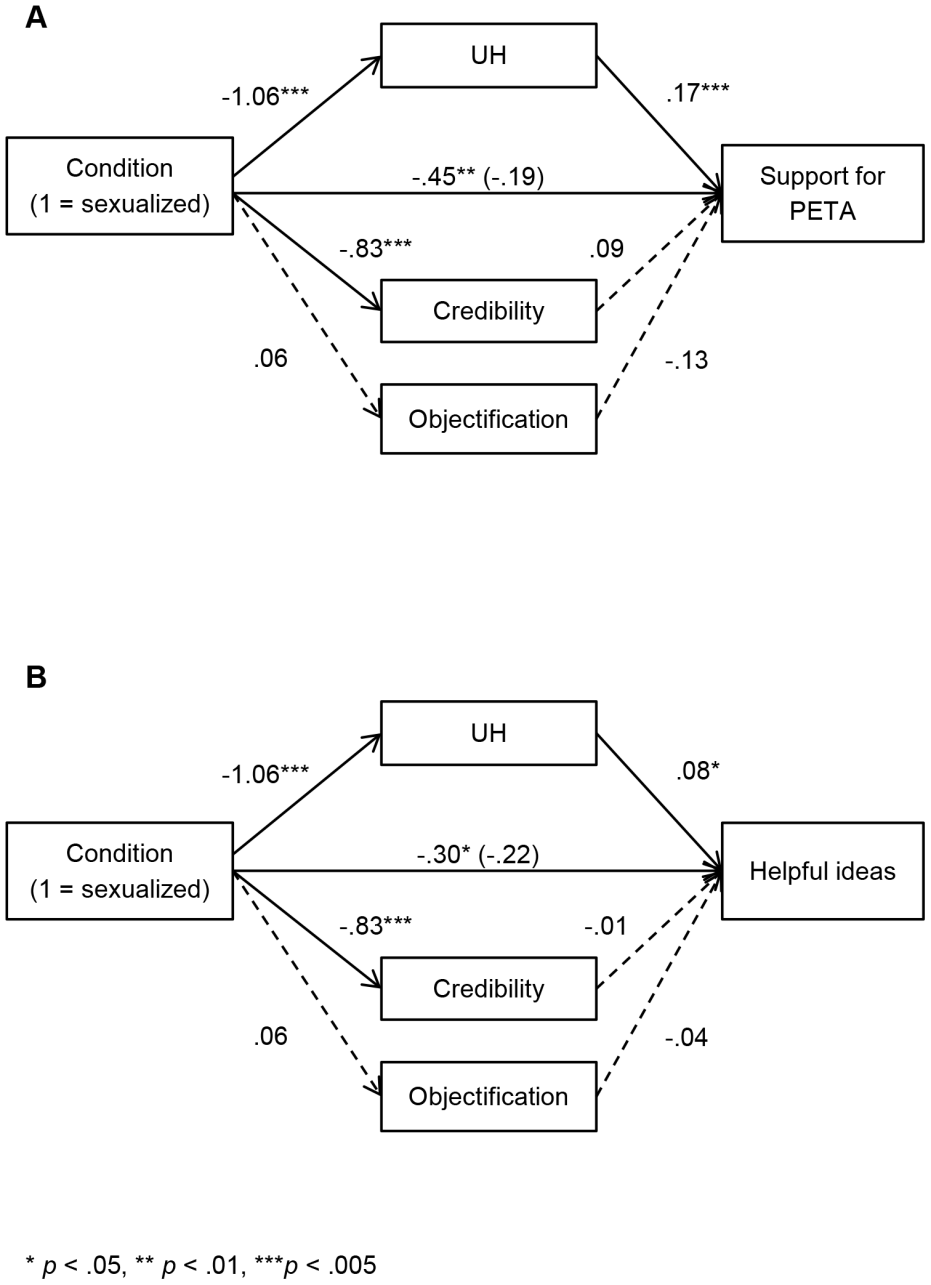
The impact of sexualized advertising on PETA support intentions and behaviors (Study 2). Mediation model showing the effect of sexualized images on (A) support for PETA and (B) ideas helpful to the animal-rights cause mediated by UH, credibility, and objectification. Solid lines represent significant paths, dashed lines represent non-significant paths.

Replicating Study 1, support for PETA was lower in the sexualized (*M* = 2.73, *SD* = 1.35), than in the non-sexualized (*M* = 3.18, *SD* = 1.43) condition, *F*(1, 276) = 5.56, *p* = .019. A main effect for participant gender was also observed, *F*(1, 276) = 30.95, *p*<.001, with women (*M* = 3.43, *SD* = 1.36) more supportive of PETA than men (*M* = 2.52, *SD* = 1.30), but there was no significant interaction between participant gender and the experimental conditions (*p* = .823). Multiple mediation analysis (Figure 2A) revealed that UH and credibility, but not objectification, were lower in the sexualized condition; however only lower UH was related to reduced support for PETA. Thus, dehumanization was the only significant mediator, with a 95% confidence interval not containing zero (UH: [−.35, −.06]; objectification: [−.07, .01]; credibility: [−.23, .04]; see Table 2 for means and correlations between items).

**Table 2:** Study 2 Means (SDs) for Non-Sexualized and Sexualized Conditions and Correlations for PETA Support, UH, Arousal and Objectification.

Study 2	Non-sexualized	Sexualized	1.	2.	3.	4.
1. PETA support	3.18 (1.43)	2.73 (1.35)	–			
2. Helpful ideas	0.96 (1.08)	0.66 (0.90)	.03	–		
3. UH	6.74 (2.01)	5.68 (2.22)	.37**	.20**	–	
4. Credibility	5.09 (1.44)	4.26 (1.53)	.30**	.13*	.64**	–
5. Objectification	2.73 (0.65)	2.79 (0.66)	−.21**	−.10	−.43**	−.35**

* *p*<.05.

** *p*<.01.

One explanation for the finding showing no difference in objectification across conditions is that most previous research has used face-ism – the size of the face relative to the body in an image – to manipulate objectification [10], [25], [28]. In contrast, the advertisements used here, while differing in the extent to which the women were sexualized, did *not* differ in face-ism (sexualized = .15, non-sexualized = .18; *t*(10) = .59, *p* = .57).

To assess effects on the behavioral measure of providing ideas to raise people's awareness and concern for animals, content analysis was performed. Five categories of responses were identified, which were categorized by two independent coders blind to the hypotheses and experimental conditions. The focal code was helpful/elaborated ideas, which represented substantive or reflective ideas to foster concern for animals (e.g., “display what goes on in slaughterhouses”). This can be contrasted with a separate code for minimal or trivial suggestions (e.g. “advertising”). The remaining codes were unrelated to raising awareness or concern, representing endorsement or criticism of PETA, or general comments. Independent coders showed very good agreement, with a Krippendorff's alpha [30] of .83, with disagreements resolved through discussion. Examples for each category are provided in Table 3, along with the average number of ideas provided across the four spaces and *t*-tests of differences between conditions.

**Table 3:** Coded Categories of Responses for How to Raise Awareness/Concern for Animals.

Coded categories	Examples	Sexualized	Non-sexualized	
		*n* = 138	*n* = 142	*t*(278)
1. Helpful/elaborated ideas	“Inform people about how to buy cruelty free products/where they are available”, “Coverage/reporting acts of animal cruelty”	.66 (.90)	.96 (1.08)	2.51*
2. Trivial/unelaborated ideas	“advertise”,“commercials”	.30 (.75)	.27 (.68)	−.26, ns
3. General comments/opinions	“Nature decide who will live or die not humans”, “Excessive animal violence leads to increased human violence”	.45 (.76)	.31 (.69)	−1.61, ns
4. Criticism of PETA	“Do not make ads like in this survey”, “At this point, you might as well just sponsor porn”	.40 (.71)	.20 (.49)	−2.76**
5. Endorsement of PETA	“more ads like this”, “more naked women print ads”	.07 (.25)	.08 (.27)	.40, ns

* *p*<.05.

** *p*<.01.

Focusing on the main category (helpful/elaborated ideas), participants in the sexualized condition generated significantly fewer helpful ideas (sexualized, *M* = .66, *SD* = .90, non-sexualized, *M* = .96, *SD* = 1.08; *F*(1, 276) = 6.12, *p* = .014), with no participant gender or interaction effects (*p*s>.301). As with support for PETA, multiple mediation (Figure 2B) revealed that only dehumanization (UH) was related to generating fewer helpful ideas after viewing the sexualized advertisements, with a 95% confidence interval not including zero (UH: [−.19, −.02]; objectification ([−.04, .01]; credibility ([−.06, .10]).

In addition to generating fewer helpful ideas, as shown in Table 3, significantly more participants in the sexualized condition spontaneously criticized PETA's advertising strategy, although the overall extent of the criticism of PETA was relatively low. For this code there were also no participant gender or interaction effects (*p*s>.365). Differences across conditions in other categories were not significant, nor were there significant participant gender or interaction effects (*p*s>.415; for analyses examining political orientation and other factors, see Analyses S2)

## General Discussion

In a crowded media marketplace, advocacy organizations promoting ethical causes struggle to gain public attention. It is understandable that they might employ “sex sells” strategies to gain men's interest and support. Yet these findings demonstrate that this approach can backfire, with exposure to sexualized advertising reducing both intentions to support the ethical organization (Studies 1 & 2) and behavior helpful to the animal-rights cause (Study 2). In both studies, conducted in different nations (Australia, the United States), and with different demographics (male undergraduates, mixed-gender community sample), consistent evidence of mediation by dehumanization indicated that the dehumanization of the women in the sexualized advertisements is central to explaining these findings.

Alternative explanations across the two studies were not supported. Study 1 showed that while men found the sexualized advertisements more arousing, arousal was unrelated to support. Study 2 showed that the lower credibility of sexualized women as advocates for the ethical cause could also not account for the findings, and nor could objectification. Mean differences for objectification revealed that women in the sexualized and non-sexualized advertisements were objectified to a similar degree. Despite this, and consistent with its theoretical link to dehumanization [10], objectification was negatively correlated with uniquely human characteristics (UH). This shows that even in the absence of mean differences in objectification, the dehumanizing effect of sexualized images of women can have a negative impact.

Overall, these findings are the first to demonstrate that sexualized images that dehumanize women reduce concern for ethical behavior in a domain *unrelated* to gender relations and sex. This extends research showing that women's dehumanization is associated with increased tolerance for unethical behavior towards women – specifically men's attitudes towards sexual harassment and rape [19]. These findings open the way for further research to explore whether similarly negative effects would occur if sexualized images of women were used to sell ethical causes other than the treatment of animals, for instance, in promoting action to address poverty.

In sum, our findings indicate that organizations promoting ethical causes should be especially concerned with communicating their message ethically, specifically in ways that do not dehumanize women. They also show that dehumanizing women not only has negative consequences for women [10], [19], but also for the ethical causes that traffic in them.

## Supporting Information

Analyses S1 Analyses examining effects of participant political orientation and age.
(DOCX)

Analyses S2 Analyses examining effects of participant political orientation and age, advertisement offensiveness, familiarity, liking and attractiveness.
(DOCX)

Appendix S1 Links to the advertisements used in the studies.
(DOCX)

Appendix S2 Full list of items.
(DOCX)
