# Supplementary material for: When Sex Doesn't Sell: Using Sexualized Images of Women Reduces Support for Ethical Campaigns
Source: PLoS One. 2013 Dec 18;8(12):e83311. doi: 10.1371/journal.pone.0083311 (PMC3867429; doi:10.1371/journal.pone.0083311)
Supplement: Analyses S1 — Analyses examining effects of participant political orientation and age. (DOCX) [file pone.0083311.s001.docx]

**Supplemental Analyses S1**

As support for PETA might be seen to be influenced by political orientation; we performed additional analyses with political orientation. Forty-three percent of the sample reported intending to vote for a right-wing party, 43% for a left-wing party, and 14% were independent/unaligned. However, including left/right wing voting intentions (coded as a dichotomous variable), revealed no interaction with condition (*p* = .975), strongly indicating that the findings were not influenced by left-wing bias typically attributed to undergraduates. Further analyses were conducted to assess effects of age – including participant age as a covariate did not affect the significance of the results.
