# Supplementary material for: When Sex Doesn't Sell: Using Sexualized Images of Women Reduces Support for Ethical Campaigns
Source: PLoS One. 2013 Dec 18;8(12):e83311. doi: 10.1371/journal.pone.0083311 (PMC3867429; doi:10.1371/journal.pone.0083311)
Supplement: Analyses S2 — Analyses examining effects of participant political orientation and age, advertisement offensiveness, familiarity, liking and attractiveness. (DOCX) [file pone.0083311.s002.docx]

**Supplemental Analyses S2**

As with Study 1, additional analyses were performed with political orientation. Twenty-three percent of the sample reported intending to vote for a right-wing party, 59% for left-wing parties, and 18% independent/unaligned. However, including left/right wing voting intentions (coded as a dichotomous variable), revealed no interaction with condition (*ps >*.59), strongly indicating that the findings were not influenced by left-wing bias. Results remained significant after including age as a covariate.

The greater proportion of criticisms of PETA in the sexualized condition suggested participants were more offended by these advertisements. The questionnaire also included an item regarding whether participants were offended by the advertisements (i.e., “Are you offended by this advertisement?”; 1 = *Not at all*, 7 = *Very much*), however, overall, levels of offence were low (sexualized, *M* = 2.30, *SD*, = 1.65; non-sexualized, *M* = 1.56, *SD* = .84). Further, offence was included in multiple mediation analyses and was found not to be a mediator, and UH remained the only significant mediator for both PETA support, [-.36, -.07], and helpful ideas [-.18, -.01]. This provides further support for the interpretation that the dehumanization of the women depicted in advertisements, not levels of offence and/or other contributing factors were driving these findings.

Including familiarity, liking of the advertisement, and attractiveness of the models made no difference to the significance of either mediation model when these were included as covariates or when included as alternative mediators.
