# Supplementary material for: When Sex Doesn't Sell: Using Sexualized Images of Women Reduces Support for Ethical Campaigns
Source: PLoS One. 2013 Dec 18;8(12):e83311. doi: 10.1371/journal.pone.0083311 (PMC3867429; doi:10.1371/journal.pone.0083311)
Supplement: Appendix S1 — Links to the advertisements used in the studies. (DOCX) [file pone.0083311.s003.docx]

**Appendix S1**

**Advertisements**

Advertisements 1-3: both Studies; Advertisements 4-6: Study 2 only. Advertisements were equalized on size (500 x 660 pixels), and obtained from the following sources:

**Sexualized.**

1. (Against animal testing) [www.mediapeta.com/peta/Images/Main/Sections/MediaCenter/PrintAds/LaylaKayleigh-ATBH.jpg](file:///C:\Users\Renata\Documents\Renata's%20documents\PETA\Psych%20Science\www.mediapeta.com\peta\Images\Main\Sections\MediaCenter\PrintAds\LaylaKayleigh-ATBH.jpg)
2. (Against using animal fur for clothing)

[www.mediapeta.com/peta/Images/Main/Sections/MediaCenter/PrintAds/CharlotteRoss_horiz_72.jpg](http://www.mediapeta.com/peta/Images/Main/Sections/MediaCenter/PrintAds/CharlotteRoss_horiz_72.jpg)

1. (Vegetarianism)

[www.mediapeta.com/peta/Images/Main/Sections/MediaCenter/PrintAds/SophieMonk_updated_72.jpg](http://www.mediapeta.com/peta/Images/Main/Sections/MediaCenter/PrintAds/SophieMonk_updated_72.jpg)

1. (Spay and neuter pets)

<http://www.mediapeta.com/peta/Images/Main/Sections/MediaCenter/PrintAds/large-SashaGrey.jpg>

1. (Against using animals in entertainment)

<http://www.mediapeta.com/peta/Images/Main/Sections/MediaCenter/kristen%20johnston_300.jpg>

1. (Vegetarianism)

<http://www.mediapeta.com/peta/Images/Main/Sections/MediaCenter/PrintAds/bonniejillPETA72.jpg>

**Non-sexualized.**

1. (Against animal testing)

<http://www.mediapeta.com/peta/images/main/sections/mediacenter/printads/laylaP2-72.jpg>

1. (Against using animal fur for clothing)

[www.mediapeta.com/peta/images/main/sections/mediacenter/printads/P2daniella72.pdf](http://www.mediapeta.com/peta/images/main/sections/mediacenter/printads/P2daniella72.pdf)

1. (Vegetarianism)

<http://www.mediapeta.com/peta/Images/Main/Sections/MediaCenter/PrintAds/AdJorjaFox.pdf>

1. (Spay and neuter pets)

<http://www.mediapeta.com/peta/Images/Main/Sections/MediaCenter/PrintAds/catpower72.pdf>

1. (Against using animals in entertainment)

[www.mediapeta.com/peta/Images/Main/Sections/MediaCenter/PrintAds/Lea_Michele_HDC_72.jpg](http://www.mediapeta.com/peta/Images/Main/Sections/MediaCenter/PrintAds/Lea_Michele_HDC_72.jpg)

1. (Vegetarianism)

<http://www.mediapeta.com/peta/Images/Main/Sections/MediaCenter/PrintAds/AdAlyssaMilano.pdf>
