# Supplementary material for: When Sex Doesn't Sell: Using Sexualized Images of Women Reduces Support for Ethical Campaigns
Source: PLoS One. 2013 Dec 18;8(12):e83311. doi: 10.1371/journal.pone.0083311 (PMC3867429; doi:10.1371/journal.pone.0083311)
Supplement: Appendix S2 — Full list of items. (DOCX) [file pone.0083311.s004.docx]

**Appendix S2**

Items used in Study 1 and Study 2

| Construct | Items |
| --- | --- |
| HU (both studies) | Overall, to what extent do you believe the people in these advertisements are: (1 = *Not at all*, 10 = *Very much*) *civilized; mature; rational; moral; complex; refined.* |
| Intentions to support PETA (both studies) | Please indicate your agreement with the following statements: (1 = *Strongly disagree*, 7 = *Strongly agree*) *If PETA sponsored a petition, I would want to sign it; I consider myself to be a supporter of PETA; I would like to participate in a group action organized by PETA, such as a march or a rally; I would like to become more involved with PETA; I do not wish to be involved with PETA in any way* (reversed, Study 2 only). |
| Arousal (Study 1) | Do you find this advertisement arousing?(1 = *Not at all*, 7 = *Very much*) |
| Credibility (Study 2) | Overall, to what extent do you believe the people in these advertisements are: (1 = *Not at all*, 7 = *Very much*) *genuine supporters of animal rights; likely to behave in ways that protect animal rights in their own lives; credible representatives for the animal rights cause; genuinely concerned about animal rights*; *likely to vote for a political candidate with a pro-animal rights agenda; likely to attend a march or rally to promote animal rights.* |
| Objectification (Study 2) | Compared to the average person, to what extent do you believe the people in these advertisements are capable of: (1 = *Much less capable*, 3 = *Equally capable*, 5 = *Much more capable*) *thought; communication; self control; planning; memory.* |
